# Activated PI3K Delta Syndrome: A Case Presentation and Literature Review

**DOI:** 10.7759/cureus.89884

**Published:** 2025-08-12

**Authors:** Angélica López Méndez, Airam A Arias Villaverde, Abraham P Sánchez López, Elizabeth Gloria Méndez, María R Hernández Zamora, Alesha Ramírez Lobato, María F Aguirre Quintero, Emilio Mondragón Rosas, José Emiliano González Flores

**Affiliations:** 1 Pediatric Nephrology, Instituto Nacional de Pediatría (INP), Mexico City, MEX; 2 School of Medicine and Health Sciences, Tecnológico de Monterrey Mexico City Campus, Mexico City, MEX; 3 Medical Genetics, Iztacalco Pediatric Hospital, Mexico City, MEX; 4 School of Medicine, Panamerican University, Mexico City, MEX; 5 Pediatric Gastroenterology and Nutrition, Instituto Mexicano del Seguro Social, Centro Médico Nacional Siglo XXI, Mexico City, MEX

**Keywords:** activated pi3k delta syndrome, genetic mutations, pi3k, pik3r1 gene, primary immunodeficiency disease

## Abstract

Activated PI3K delta syndrome (APDS) is a rare primary immunodeficiency caused by gain-of-function (GOF) mutations in *PIK3CD* or *PIK3R1*, leading to immune dysregulation. It typically presents with recurrent respiratory infections, lymphoproliferation, autoimmunity, and increased risk of malignancy. Although gastrointestinal involvement has been described, it is less frequent and often underrecognized. To our knowledge, this is the first genetically confirmed case of APDS in Mexico, highlighting an atypical presentation with significant gastrointestinal symptoms. This case underscores the challenges in diagnosing APDS in settings with limited access to genetic testing and emphasizes the importance of early recognition in patients with complex infectious and inflammatory histories.

A four-year-old male presented with chronic diarrhea, failure to thrive, and recurrent respiratory infections since infancy. Clinical evaluation revealed hepatosplenomegaly, generalized lymphadenopathy, severe malnutrition, and infections with *Pseudomonas aeruginosa*, *Clostridium difficile,* and parainfluenza virus. Endoscopic findings included malakoplakia and *Helicobacter pylori* gastritis. A multigene panel identified a heterozygous pathogenic variant in *PIK3CD* (c.3061G>A, p.Glu1021Lys), confirming APDS1. Additionally, a risk variant in *NOD2* was detected, which may have contributed to the gastrointestinal phenotype. The patient was managed with immunoglobulin replacement, antimicrobial prophylaxis, and multidisciplinary care, leading to clinical improvement.

This case illustrates the variable presentation of APDS and highlights the importance of including primary immunodeficiencies in the differential diagnosis of children with recurrent infections and gastrointestinal manifestations. Early genetic confirmation facilitates tailored management and improves outcomes. Future research should explore genotype-phenotype correlations, especially in patients with multiple genetic variants, and assess the long-term benefits of targeted therapies such as PI3Kδ inhibitors.

## Introduction

Activated PI3K delta syndrome (APDS), also known as p110δ-activating mutation causing senescent T cells, lymphadenopathy, and immunodeficiency (PASLI), is a rare primary immunodeficiency first described in 2013 [1-4]. Although the exact prevalence remains uncertain, initial reports estimated it to be approximately one in 1,000,000 live births. However, this may vary across populations and likely underrepresents the true burden due to limited genetic screening [1]. APDS is caused by gain-of-function (GOF) mutations in the *PIK3CD* gene, located on chromosome 1p36, which encodes the catalytic subunit p110δ of phosphoinositide 3-kinase delta (PI3Kδ) (APDS1), or in the *PIK3R1* gene, which encodes the regulatory subunit p85α (APDS2) [2]. These mutations result in hyperactivation of the PI3K/AKT/mTOR pathway, which is predominantly expressed in leukocytes, disrupting key cellular processes such as growth, survival, proliferation, and differentiation of T and B lymphocytes [5-7].

Clinically, APDS is characterized by recurrent infections, non-malignant lymphoproliferation (such as lymphadenopathy and hepatosplenomegaly), autoimmune manifestations (mainly cytopenias), and an increased risk of lymphoma [1,6-8]. Immunological dysfunction includes CD8+ T-cell senescence, elevated transitional B cells, reduced class-switched memory B cells, and frequently a hyper-IgM phenotype with decreased IgG and IgA levels [5,6,8]. These features can resemble common variable immunodeficiency (CVID) or hyper-IgM syndrome; however, APDS often presents earlier in life and is distinguished by prominent non-malignant lymphoproliferation (e.g., generalized lymphadenopathy, hepatosplenomegaly) and elevated IgM levels despite normal or near-normal IgG. A precise differential diagnosis is essential due to the availability of targeted therapies such as mTOR inhibitors (e.g., sirolimus) and selective PI3Kδ inhibitors (e.g., leniolisib) [1,5,7,9].

Despite advances in the global characterization of APDS, reports from Latin America, particularly Mexico, are currently nonexistent. To our knowledge, this case represents the first genetically confirmed report of APDS in Mexico, highlighting its relevance in raising clinical awareness and improving access. 

## Case presentation

A four-year-old male was evaluated at a tertiary care center with a history of gastrointestinal symptoms beginning at four months of age, including regurgitation, vomiting, abdominal distension, and poor weight gain. At six months, he developed persistent diarrhea, and by age two, he began experiencing recurrent upper respiratory tract infections. He was admitted with dysenteric stools, unintentional weight loss, night sweats, and a high-grade fever responsive to antipyretics. On physical examination, the patient exhibited signs of malnutrition, cachexia, mucocutaneous pallor, generalized lymphadenopathy (submandibular, cervical, axillary, and inguinal), and hepatosplenomegaly. An oncologic process was ruled out.

At a referral hospital, he was diagnosed with gastroenteritis due to *Pseudomonas aeruginosa*, which was resolved after treatment. He subsequently developed a *Clostridium difficile* infection managed with oral vancomycin. Due to poor clinical progression, he was referred back to the tertiary center, where further workup revealed lower gastrointestinal bleeding, bicytopenia (anemia and thrombocytopenia), and a lymphoproliferative syndrome (hepatosplenomegaly and generalized lymphadenopathy).

During hospitalization, the patient developed severe pneumonia caused by the parainfluenza virus, requiring invasive mechanical ventilation and admission to the intensive care unit for mixed shock (septic and hypovolemic), which was managed with intravenous crystalloids and vasopressors. He required multiple transfusions of packed red blood cells. The initial laboratory, immunological, and serological assessments are summarized in Table 1. 

**Table 1:** Summary of initial laboratory, immunological, and serological assessments. Results support immunodeficiency with selective IgM deficiency and past exposure to several viral agents, without hematologic involvement. HBsAg: hepatitis B surface antigen; HCV: hepatitis C virus

Section	Test/Parameter	Result
Immunological profile	IgM	Decreased
	IgA and IgG	Within normal range
Tumor markers	General panel	Negative
Serology	HIV, HBsAg, anti-HCV, toxoplasma	Non-reactive
	Herpes virus 1 and 2	IgG positive, IgM negative
	Cytomegalovirus	IgG positive, IgM negative
	Rubella	IgG and IgM positive
Bone marrow aspiration	Morphology	No hematological abnormalities

The patient was managed through a multidisciplinary approach involving gastroenterology, immunology and allergy, internal medicine, infectious diseases, nephrology, and mental health services. Treatment included monthly intravenous immunoglobulin (IVIG), antimicrobial prophylaxis with a third-generation quinolone, multivitamin supplementation, and hematinics. The clinical course was favorable, with good oral tolerance, normalization of bowel movements, gradual weight gain, and no further infections or hospital readmissions.

The medical genetics team, after obtaining informed consent, performed molecular studies using a multigene panel for primary immunodeficiencies. The results revealed a heterozygous pathogenic variant in the *PIK3CD* gene (c.3061G>A, p.Glu1021Lys), a heterozygous risk allele in the *NOD2* gene (c.3019dup, p.Leu1007Profs*2), which has been previously associated with Crohn's disease and immune dysregulation [9], and a benign pseudodeficiency variant in the *GALC* gene (c.16805T>C, p.Ile562Thr). A diagnosis of APDS1 was established (Table 2). Given the patient’s clinical improvement, he was discharged with multidisciplinary follow-up.

**Table 2:** Results from a multigene panel for primary immunodeficiencies. A pathogenic variant in *PIK3CD* confirmed the diagnosis of activated PI3K delta syndrome type 1 (APDS1). The concurrent presence of a *NOD2* risk allele may explain the patient’s prominent gastrointestinal manifestations, while the *GALC* variant was classified as benign and clinically irrelevant in this context.

Gene	Variant	Zygosity	Clinical	Comments
PIK3CD	c.3061G>A (p.Glu1021Lys)	Heterozygous	Pathogenic	Confirms diagnosis of activated PI3K delta syndrome type 1 (APDS1)
NOD2	c.3019dup (p.Leu1007Profs*2)	Heterozygous	Risk allele	May contribute to GI phenotype via immune dysregulation
GALC	c.16805T>C (p.Ile562Thr)	Heterozygous	Benign (Pseudodeficiency allele)	Not clinically relevant in the current presentation

## Discussion

This case describes a four-year-old male patient with a complex clinical course beginning at four months of age with persistent gastrointestinal symptoms, followed by recurrent respiratory infections starting at age two, severe malnutrition, cachexia, generalized lymphadenopathy, hepatosplenomegaly, and multiple infectious complications, including *Pseudomonas aeruginosa* gastroenteritis, *Clostridium difficile* infection, and severe pneumonia caused by parainfluenza virus. Molecular analysis confirmed the presence of a pathogenic variant in the *PIK3CD* gene (c.3061G>A, p.Glu1021Lys), establishing the diagnosis of APDS1. This case contributes to the international medical literature by documenting an unusually severe phenotype, characterized by predominant gastrointestinal manifestations and susceptibility to atypical opportunistic pathogens. It also emphasizes the importance of genetic diagnosis even in resource-limited settings such as Mexico.

Although the patient’s phenotype shares common features with other reported APDS1 cases, such as recurrent respiratory infections and non-malignant lymphoproliferation [3,10], the severity and early onset of gastrointestinal symptoms, including persistent diarrhea, intestinal malakoplakia, and *Helicobacter pylori* gastritis, are uncommon. Studies, such as Maccari et al., report that severe gastrointestinal involvement is infrequent in APDS1, with respiratory infections and bronchiectasis being more typical [11]. Similarly, Jamee et al., in a systematic review of 243 APDS patients, reported gastrointestinal manifestations in only 26.7% of cases, reinforcing their relative rarity and frequent underrecognition [5]. Although malakoplakia has been reported in other immunocompromised states, including primary immunodeficiencies and transplant recipients, its occurrence in APDS has not been previously described, suggesting profound immune dysregulation possibly mediated by hyperactivation of the PI3K/AKT/mTOR pathway [7,4,11,12]. The co-occurrence of *Pseudomonas* infection and mixed shock secondary to viral pneumonia further supports the hypothesis of a broader immunodeficiency spectrum than traditionally described, given that the literature typically highlights herpesvirus susceptibility as a hallmark of APDS [4,13,14].

Compared to international cohorts, such as that analyzed by Elkaim et al., which included 51 APDS patients, this case presents important divergences [4]. While bronchiectasis is reported in 60% of cases, it was absent in our patient, possibly due to younger age at diagnosis or geographic, environmental, or genetic differences [12,13]. The severe lymphoproliferation observed, requiring evaluation to rule out lymphoma, represents an intense but disease-consistent manifestation. An additional relevant finding was the presence of a risk allele in the *NOD2* gene (c.3019dup), whose synergistic interaction with the *PIK3CD* mutation may exacerbate gastrointestinal inflammation. This raises the possibility of an additional genetic contribution not yet explored in prior cohorts [10]. This observation supports the hypothesis that modifier variants may influence APDS1 phenotypic expression in Latin American populations, representing a promising avenue for future research.

In contrast to APDS2 (caused by *PIK3R1* mutations), this case highlights several distinguishing features. Patients with APDS2 tend to show a higher risk of lymphoma (25% vs. 13% in APDS1) and growth delay, both absent in our patient [12,15]. Nonetheless, phenotypic overlap between APDS1 and APDS2 remains significant, including recurrent infections and lymphoproliferation [3]. Individual reports, such as those by Maccari et al., emphasize severe viral infections in APDS1, although specific findings in our case, such as intestinal malakoplakia and parainfluenza pneumonia, are rare and expand the known clinical spectrum [11,14].

The patient was managed with IVIG, antimicrobial prophylaxis, and multidisciplinary support, showing a favorable clinical response. This approach is consistent with current literature, where approximately 30% of APDS patients receive immunoglobulin therapy to prevent infections [4]. However, targeted therapies, such as mTOR inhibitors (e.g., sirolimus) or selective PI3Kδ inhibitors (e.g., leniolisib), were not used, likely due to limited local access. While sirolimus has demonstrated efficacy in reducing lymphoproliferation in ~22% of cases, leniolisib, recently approved, has shown benefits in reducing lymphadenopathy and improving immunological markers in clinical trials [14]. In a phase 3 clinical trial, Rao et al. reported that leniolisib was well tolerated and showed significant improvement over placebo in both primary endpoints, reducing lymphadenopathy and increasing the percentage of naïve B cells, reflecting a favorable effect on the immune dysregulation and deficiency seen in APDS [16]. The lack of access to such treatments in this case highlights a critical gap in innovative therapy availability in middle-income countries like Mexico.

This case also proposes valuable future research directions. One is the evaluation of the frequency and variability of *PIK3CD* and *PIK3R1* mutations in Mexican populations, where genomic research in primary immunodeficiencies remains limited. The co-occurrence of an *NOD2* risk allele further supports the need to investigate modifier genes and their role in APDS expression. Similar modifier gene effects have been described in other primary immunodeficiencies, such as *DOCK8* deficiency and *STAT1* GOF mutations, where additional genetic variants contribute to phenotypic variability and disease severity. Additionally, identifying progression biomarkers, such as S6K phosphorylation in lymphocytes, an indicator of mTOR hyperactivation, could enhance patient monitoring and personalized therapy [8,10,17,18]. Longitudinal studies documenting atypical manifestations, such as intestinal malakoplakia, or assessing the efficacy of PI3Kδ inhibitors in patients with severe gastrointestinal involvement, may significantly advance understanding of this disease. Major challenges in studying and managing such patients in Mexico include the scarcity of reported cases, lack of long-term follow-up, and absence of systematic genetic screening, which hinder the identification of atypical phenotypes and the evaluation of therapeutic outcomes. Moreover, access to innovative therapies like leniolisib remains limited in many middle-income countries, reducing clinical management options.

This case provides a valuable contribution to the understanding of APDS1 by documenting a severe and atypical phenotype with early gastrointestinal manifestations, susceptibility to uncommon opportunistic pathogens, and previously undescribed inflammatory findings. It underscores the importance of implementing genetic testing for primary immunodeficiencies, even in resource-constrained settings, and the urgent need to improve access to targeted therapies. Furthermore, it highlights the value of expanding genetic research in Latin American populations to characterize local variants and enhance patient care in the region.

## Conclusions

APDS poses significant diagnostic and therapeutic challenges in pediatrics, particularly in settings where clinical recognition and access to molecular testing are limited. This case highlights the importance of considering primary immunodeficiencies in the differential diagnosis of complex clinical presentations involving recurrent infections, atypical lymphoproliferation, and autoimmune manifestations from early childhood.

The identification of a pathogenic variant in the *PIK3CD* gene not only enabled a definitive diagnosis but also guided a more precise and multidisciplinary management approach, resulting in favorable clinical outcomes. To our knowledge, as the first genetically confirmed case of APDS reported in Mexico, this case underscores the need to strengthen clinical suspicion, expand access to genetic testing, and promote comprehensive care for patients with suspected primary immunodeficiencies. These efforts will ultimately contribute to improved recognition and management of rare diseases across Latin America.

## References

[1] Singh A, Joshi V, Jindal AK, Mathew B, Rawat A (2020). An updated review on activated PI3 kinase delta syndrome (APDS). Genes Dis.

[2] Crisanto-López IE, Pérez-Arzola AA, Hernández-Castañeda Y (2024). Case report on activated PI3K-delta syndrome. Bol Med Hosp Infant Mex.

[3] Aghamohamadi N, Mehrabadi AZ (2020). Activated PI3K-delta syndrome: pathogenesis, clinical manifestations, diagnosis, classification, and management. Immunol Genet J.

[4] Elkaim E, Neven B, Bruneau J (2016). Clinical and immunologic phenotype associated with activated phosphoinositide 3-kinase δ syndrome 2: a cohort study. J Allergy Clin Immunol.

[5] Jamee M, Moniri S, Zaki-Dizaji M (2020). Clinical, immunological, and genetic features in patients with activated PI3Kδ syndrome (APDS): a systematic review. Clin Rev Allergy Immunol.

[6] Deau MC, Heurtier L, Frange P (2014). A human immunodeficiency caused by mutations in the PIK3R1 gene. J Clin Invest.

[7] Lucas CL, Chandra A, Nejentsev S, Condliffe AM, Okkenhaug K (2016). PI3Kδ and primary immunodeficiencies. Nat Rev Immunol.

[8] Maccari ME, Wolkewitz M, Schwab C (2023). Activated phosphoinositide 3-kinase δ syndrome: update from the ESID Registry and comparison with other autoimmune-lymphoproliferative inborn errors of immunity. J Allergy Clin Immunol.

[9] Kayali S, Fantasia S, Gaiani F, Cavallaro LG, de'Angelis GL, Laghi L (2025). NOD2 and Crohn’s disease clinical practice: from epidemiology to diagnosis and therapy, rewired. Inflamm Bowel Dis.

[10] Michalovich D, Nejentsev S (2018). Activated PI3 kinase delta syndrome: from genetics to therapy. Front Immunol.

[11] Maccari ME, Abolhassani H, Aghamohammadi A (2018). Disease evolution and response to rapamycin in activated phosphoinositide 3-kinase δ syndrome: the European Society for Immunodeficiencies registry. Front Immunol.

[12] Crank MC, Grossman JK, Moir S (2014). Mutations in PIK3CD can cause hyper IgM syndrome (HIGM) associated with increased cancer susceptibility. J Clin Immunol.

[13] Brodsky NN, Lucas CL (2021). Infections in activated PI3K delta syndrome (APDS). Curr Opin Immunol.

[14] Dornan GL, Siempelkamp BD, Jenkins ML, Vadas O, Lucas CL, Burke JE (2017). Conformational disruption of PI3Kδ regulation by immunodeficiency mutations in PIK3CD and PIK3R1. Proc Natl Acad Sci U S A.

[15] De SK (2024). Leniolisib: a novel treatment for activated phosphoinositide-3 kinase delta syndrome. Front Pharmacol.

[16] Rao VK, Webster S, Šedivá A (2023). A randomized, placebo-controlled phase 3 trial of the PI3Kδ inhibitor leniolisib for activated PI3Kδ syndrome. Blood.

[17] Ravendran S, Hernández SS, König S, Bak RO (2022). CRISPR/Cas-based gene editing strategies for DOCK8 immunodeficiency syndrome. Front Genome Ed.

[18] Giovannozzi S, Demeulemeester J, Schrijvers R, Gijsbers R (2021). Transcriptional profiling of STAT1 gain-of-function reveals common and mutation-specific fingerprints. Front Immunol.

